# Biofilm Formation Mechanisms of *Pseudomonas aeruginosa* Predicted via Genome-Scale Kinetic Models of Bacterial Metabolism

**DOI:** 10.1371/journal.pcbi.1004452

**Published:** 2015-10-02

**Authors:** Francisco G. Vital-Lopez, Jaques Reifman, Anders Wallqvist

**Affiliations:** Department of Defense Biotechnology High Performance Computing Software Applications Institute, Telemedicine and Advanced Technology Research Center, U.S. Army Medical Research and Materiel Command, Fort Detrick, Maryland, United States of America; The Pennsylvania State University, UNITED STATES

## Abstract

A hallmark of *Pseudomonas aeruginosa* is its ability to establish biofilm-based infections that are difficult to eradicate. Biofilms are less susceptible to host inflammatory and immune responses and have higher antibiotic tolerance than free-living planktonic cells. Developing treatments against biofilms requires an understanding of bacterial biofilm-specific physiological traits. Research efforts have started to elucidate the intricate mechanisms underlying biofilm development. However, many aspects of these mechanisms are still poorly understood. Here, we addressed questions regarding biofilm metabolism using a genome-scale kinetic model of the *P*. *aeruginosa* metabolic network and gene expression profiles. Specifically, we computed metabolite concentration differences between known mutants with altered biofilm formation and the wild-type strain to predict drug targets against *P*. *aeruginosa* biofilms. We also simulated the altered metabolism driven by gene expression changes between biofilm and stationary growth-phase planktonic cultures. Our analysis suggests that the synthesis of important biofilm-related molecules, such as the quorum-sensing molecule *Pseudomonas* quinolone signal and the exopolysaccharide Psl, is regulated not only through the expression of genes in their own synthesis pathway, but also through the biofilm-specific expression of genes in pathways competing for precursors to these molecules. Finally, we investigated why mutants defective in anthranilate degradation have an impaired ability to form biofilms. Alternative to a previous hypothesis that this biofilm reduction is caused by a decrease in energy production, we proposed that the dysregulation of the synthesis of secondary metabolites derived from anthranilate and chorismate is what impaired the biofilms of these mutants. Notably, these insights generated through our kinetic model-based approach are not accessible from previous constraint-based model analyses of *P*. *aeruginosa* biofilm metabolism. Our simulation results showed that plausible, non-intuitive explanations of difficult-to-interpret experimental observations could be generated by integrating genome-scale kinetic models with gene expression profiles.

## Author Summary

*Pseudomonas aeruginosa* is one of the most frequently found bacterial pathogens in patients with chronic infections, such as chronic wounds and cystic fibrosis. The persistence of *P*. *aeruginosa* in these infections is enabled by its ability to form biofilms. Standard antibiotic treatments, effective against bacteria living as single cells, are generally unsuccessful against biofilms. Understanding the physiological adaptations of bacterial biofilms will make it possible to design specific countermeasures and treatments for biofilm-based persistent infections. In this study, we investigated mechanisms underlying biofilm formation using a genome-scale kinetic model of the *P*. *aeruginosa* metabolic network and gene expression profiles. Our analysis provides insights on how *P*. *aeruginosa* regulates its metabolism to synthesize molecules that are important for biofilm formation. Based on these insights, we predicted new enzymatic reactions that can be targeted to develop anti-biofilm drugs. Notably, our approach can be used to characterize important metabolic mechanisms and strategies adapted by pathogenic bacteria refractory to conventional antibiotic treatments.

## Introduction

*Pseudomonas aeruginosa* is one of the most frequently found bacteria in chronic wounds and in the lungs of cystic fibrosis patients, where its ability to create opportunistic infections is potentiated by forming and maintaining biofilms [1]. Biofilms are communities of microorganisms that sustain themselves in a self-produced matrix of biopolymers (e.g., DNA, proteins, and polysaccharides) and exhibit a different physiology from that of planktonic (free-living) cells. Bacterial biofilms are often involved in chronic infections, where they can elicit recurring and persisting pathologies. Bacteria living in biofilms are less susceptible to the inflammatory and immune responses of their host and are considerably more resistant to antibiotic treatment than planktonic bacteria and, consequently, are more difficult to eradicate [1–3]. Thus, considerable efforts have been directed towards understanding mechanisms of biofilm development. Experimental analyses have identified several genetic components involved in initiation, growth, and maturation of biofilms [4], antibiotic resistance [5], and evasion of the host defenses [6] as contributing factors to the persistence of *P*. *aeruginosa* infections.

Hampering the understanding of the physiological differences and driving forces between cells in bacterial biofilms and planktonic cells is that these systems are greatly dependent on the experimental and environmental conditions of a particular study. Thus, it is not surprising that there is little overlap among the sets of differentially expressed genes between biofilm and planktonic cells of *P*. *aeruginosa* identified in different studies [7–11]. In order to consistently interpret complex genomic and metabolic data across altered metabolic states associated with biofilm formation, we need to use a systems biology approach that has the capacity to model and account for metabolic capabilities emerging from the enzymatic ensemble encoded by a bacterial genome [12–17]. Previous theoretical studies on *P*. *aeruginosa* metabolism have used the original genome-scale network construction [18] to interpret the time-evolving metabolic states of *P*. *aeruginosa* in cystic fibrosis [13], to predict essential genes for biofilm formation [19, 20], and as an exemplar system for simulating biofilm growth using agent-based models [21]. The latter biofilm-related works rely mainly on biomass growth for the prediction of metabolic genes that are essential for biofilm formation. In particular, Sigurdsson et al. used flux balance analysis (FBA) to predict genes essential for biomass growth under planktonic and biofilm conditions [19]. However, this approach identified the same genes as essential under both planktonic and biofilm conditions; i.e., no essential genes were predicted to be specific for the biofilm condition. As drug treatment is a potential inducer of biofilm formation, Xu et al. [20] used a different approach to prioritize the FBA-predicted essential genes based on avoidance of inducing biofilm formation. They gauged whether the inhibition of an essential gene would increase the flux through reactions that had been determined experimentally to be associated with *P*. *aeruginosa* biofilm formation [22], under the assumption that increased flux through such reactions would induce biofilm formation. They determined that inhibition of the bulk (132 out of 136) of the FBA-predicted essential genes would *induce* biofilm formation and, therefore, only four of these genes could be considered as potential drug targets.

In order to address these limitations in both experimental interpretations and theoretical studies, we developed an augmented genome-scale kinetic metabolic network model of *P*. *aeruginosa* that incorporates all previous metabolic reactions, as well as biofilm-specific metabolic pathways. This model allows for context-dependent modeling of the *P*. *aeruginosa* metabolism based on commensurate metabolic fluxes and gene expression data. We used the model to address three questions regarding *P*. *aeruginosa* metabolism under biofilm growth. First, we defined a function based on predicted metabolite concentration changes to score the propensity of inhibiting a reaction to reduce biofilm formation. This function partially incorporates the complexity of biofilm regulation reflected in the diversity of experimentally determined mutants with altered biofilm phenotype [22]. With the scoring function, we predicted 126 reaction inhibitions that reduce biofilm formation. We further prioritized this list by selecting reactions that were associated with mutants with decreased drug tolerance or attenuated virulence.

Then, we characterized metabolic fluxes and metabolite concentrations that were different between the biofilm and planktonic phenotypes. For this purpose, we used the gene expression profiles from Costaglioli et al. [11], which were obtained for biofilm and planktonic cultures grown in similar conditions and had a number of mutants tested for biofilm formation based on the hypothesis derived from the gene expression data. Our analysis of the metabolic network model provided a means by which we could provide novel and alternative explanations to the experimental results. For example, Costaglioli et al. [11] found only a few metabolic genes up-regulated in *P*. *aeruginosa* biofilms compared to stationary growth-phase planktonic cells (stationary cultures). These genes were mostly involved in the synthesis of the siderophore pyochelin and the degradation of anthranilate, a precursor of the *Pseudomonas* quinolone signal (PQS) molecule. In addition to the expected increased anthranilate degradation and pyochelin synthesis fluxes, we predicted that the synthesis of two biofilm-related molecules, PQS and the exopolysaccharide Psl, was altered in biofilms compared to stationary cultures, even though the expression of the genes associated with their synthesis did not significantly change. Finally, we explored the effects of blocking anthranilate degradation in *P*. *aeruginosa* biofilms. Costaglioli et al. [11] found that mutations of the genes involved in anthranilate degradation reduce biofilm formation and hypothesized that this was caused by a reduction in energy production. Our simulation results suggested that inhibition of anthranilate degradation had a limited impact on energy production, but caused a considerable perturbation in the synthesis of biofilm-related metabolites (including PQS). Thus, the metabolic network analysis pointed to an alternative explanation in terms of altered synthesis of secondary metabolites, such as PQS, as the primary cause of the reduction in biofilm formation.

## Results and Discussion

### Model construction and validation

We used our previously developed modeling framework [17] to construct a genome-scale condition-specific kinetic model of the *P*. *aeruginosa* metabolic network based on the metabolic network reconstruction developed by Oberhardt et al. [18]. We augmented the model by including pathways of relevant biofilm-related molecules, including anthranilate, the exopolysaccharides Psl and Pel, pyochelin, and modified lipopolysaccharides. The model had 876 genes, 685 reactions, and 504 metabolites, all of which were active under the experimental conditions considered [11]. The list of reactions and metabolites in the model is provided in S1 Supporting Information.

We provided the details for the derivation of the kinetic expressions in Materials and Methods and the kinetic model in S2 Supporting Information. An advantage of the proposed kinetic expressions is that the model parameters, namely, a reference flux distribution and gene expression ratios, can be directly derived or estimated from experimental data. In the present study, we computed the gene expression ratios between biofilm and planktonic cultures from the gene expression data from the study by Costaglioli et al. [11]. However, experimental measurements (e.g., uptake and secretion rates of extracellular metabolites) to determine an accurate reference flux distribution were not available. Thus, instead of using a single reference flux distribution, we randomly sampled the space of feasible flux distributions to generate an ensemble of reference flux distributions for each simulation condition, and then carried out the simulations for each member of the ensemble (see Materials and Methods and S1 Text). Although this parameter space is large, the mass balance, thermodynamic, and simulation condition constraints considerably reduced the size of the parameter space. This allowed us to obtain reproducible estimates of the distributions of the predicted metabolite concentration and flux changes with a relatively small sample size (i.e., 100 random reference flux distributions), as we discussed in S1 Text.

Note that the proposed model, or any kinetic model, is valid only under reasonably small changes in gene expression because the model parameters related to enzyme abundance are fixed once they are estimated for a given condition (excepts for models that incorporate gene regulatory networks, but such models are generally small). In our approach, we created a new model to simulate other conditions with different gene expressions profiles by re-estimating the related model parameters using gene expression data. Thus, in practice, we do not use a single model to simulate the difference between stationary and biofilm cultures, but a specifically parameterized model for each condition. For the simulations used to probe the inhibition of a reaction, gene expression data were not available. Thus, we carried out these simulations assuming constant gene expression levels and considered that the predicted response was indicative of the actual response, which may involve large gene expression changes. Therefore, we expect these simulations to be less accurate than the simulation of the metabolic differences between stationary and biofilm cultures.

We validated the constructed kinetic model by predicting essential reactions that were overlooked by FBA under exponential planktonic conditions. First, we predicted essential reactions using FBA (S1 Table). The FBA-based approach yielded a sensitivity of 0.60 and an accuracy of 0.76 (see S1 Text), which were in line with previous FBA results of the *P*. *aeruginosa* metabolic network based on the inhibition of single genes instead of reactions [18, 19]. Next, we used the kinetic model to simulate the effect of blocking the FBA-predicted non-essential reactions on the biomass growth rate for exponential cultures (see Materials and Methods for simulation details). Then, we ranked the reactions according to the change in the biomass growth rate caused by their inhibition (the lower the biomass growth rate, the higher the rank). Six of the predicted top 20 reactions were associated with genes that were in the set of experimentally determined essential genes for biomass growth (referred to just as *essential genes* hereafter) compiled by Sigurdsson et al. [19] (see S2 Table). The likelihood of randomly selecting six or more out of 20 reactions associated with essential genes in the model is 0.04 (see S1 Text). This result shows that our approach was able to identify important reactions for biomass growth in exponential cultures even if they were predicted to be non-essential by FBA.

### Metabolite concentration changes indicative of biofilm formation

We tested different approaches to predict reactions whose inhibition would reduce biofilm formation. Two of them were based on the effect of reaction inhibitions on the biomass growth rate or on the synthesis rate of the major components of the *P*. *aeruginosa* biofilms (i.e., the exopolysaccharides Psl and Pel, DNA, and proteins). We found that such approaches were not good predictors of mutants with altered biofilm phenotype (see S1 Text). This result, together with the observation that most of the experimentally determined mutants that have an altered biofilm phenotype [22] were associated with reactions that do not have a direct or obvious involvement in the synthesis of the major biofilm components, highlights the complexity of the underlying physiology of biofilm formation. In order to partially consider this complexity, we defined a scoring function based on the predicted metabolite concentration changes of known mutants with altered biofilm phenotype [22].

The basic idea was to use a set of metabolic reactions known to be associated with genes that either reduce or increase biofilm formation (S3 Table) [22] and link the predicted metabolite concentration changes caused by the inhibition these reactions to biofilm formation. For simplicity, we referred to the metabolic reactions associated with genes whose mutation reduced or increased biofilm formation as biofilm-reducing or biofilm-increasing reactions, respectively. The metabolites were classified in four sets according to the predicted concentration changes. Sets 1 and 2 included metabolites that increased or decreased, respectively, when inhibiting biofilm-reducing reactions, whereas sets 3 and 4 included metabolites that increased or decreased, respectively, when inhibiting biofilm-increasing reactions. Then, for the inhibition of a metabolic reaction, the scoring function counts how many metabolite concentration changes were common to the inhibition of biofilm-reducing and biofilm-increasing reactions. If the inhibition of the reaction had more metabolite concentration changes in common with the biofilm-reducing reactions than with the biofilm-increasing reactions, then inhibiting this reaction was predicted to reduce biofilm formation, and vice versa. Note that for this analysis, in addition to the non-essential reactions, we included the inhibition of essential reactions by multiplying their reaction rate by a factor of 0.01. See Materials and Methods for a detailed explanation of the scoring function and the metabolite sets and S1 Text for the validation of this approach.

We added 15 additional low biofilm-producing mutants that we found in the literature [23–25] (S4 Table) to the set identified by Musken et al. [22] to define the metabolite concentration changes that were specific to the inhibition of either biofilm-reducing or biofilm-increasing reactions. S5 Table lists the predicted metabolites for each set. We did not find evidence in the literature to support the predicted metabolite sets, except for one case. Musken et al. [22] identified the low biofilm-producing mutant *hutU* (PA5100) and the high biofilm-producing mutants *hisC*1 (PA4447), *hisD* (PA4448), *hutH* (PA5098), and PA0006. We predicted that urocanate was depleted in these high biofilm-producing mutants and accumulated in the *hutU* mutant. Urocanate is an effector of the transcriptional regulator *hutC* that represses the transcription of the *hut* genes, including itself. The protein hutC is bound to the promoters of the *hut* genes when the concentration of urocanate is low, and it disassociates when urocanate accumulates, freeing the promoters for transcription [26]. Thus, the transcription of *hutC* is expected to be low in these high biofilm producers. This is in line with the high biofilm-producing phenotype of a *hutC* transposon mutant reported by Yeung et al. [27], although the mechanism by which *hutC* mutation promotes biofilm formation is unknown.

The metabolites in sets 1 to 4 were scattered across the metabolic network. Of the 95 metabolic pathways in the model (as defined in the KEGG database, http://www.genome.jp/kegg/), 73 pathways included at least one metabolite. However, some pathways had a relatively high number of these metabolites; S6 Table lists the metabolic pathways with five or more metabolites from each metabolite set. The metabolites whose concentration increased when inhibiting biofilm-decreasing reactions (set 1) appeared in higher number in the pathways of valine, leucine, and isoleucine degradation; arginine and proline metabolism; cysteine and methionine metabolism; and glyoxylate and dicarboxylate metabolism. Fatty acid biosynthesis was the only pathway with more than five metabolites whose concentration decreased when inhibiting biofilm-reducing reactions (set 2), whereas the pentose phosphate pathway was the only one with five or more metabolites whose concentration increased when inhibiting biofilm-increasing reactions (set 3). The metabolites that decreased in biofilm-increasing reactions (set 4) appeared more frequently in the pathways of cysteine and methionine metabolism; glycine, serine, and threonine metabolism; and methane, pyruvate, and fatty acid metabolism.

We observed a considerable overlap between the sets of metabolites that increased when inhibiting biofilm-reducing reactions (set 1) and decreased when inhibiting biofilm-increasing reactions (set 4), sharing 26 metabolites. These 26 metabolites frequently appeared in the methane metabolism, fatty acid metabolism, glycolysis/gluconeogenesis, cysteine and methionine metabolism, glyoxylate metabolism, and butanoate metabolism. We believe that these metabolites are reasonable candidates for having a role in the regulation of biofilm formation and provide leads for further studies to understand the physiology of bacterial biofilms.

### Predicted target reactions to reduce biofilm formation

We found 126 and 113 reactions whose inhibitions were predicted to decrease and increase biofilm formation, respectively. S7 Table provides the complete lists of these reaction sets. Reactions whose inhibition decreased biofilm formation frequently appeared in the fatty acid biosynthesis pathway, arginine and proline metabolism, cysteine and methionine metabolism, valine, leucine, and isoleucine degradation, and butanoate metabolism. In contrast, the reactions that increased biofilm formation frequently appeared in the amino sugar and nucleotide sugar metabolism, peptidoglycan biosynthesis, pentose phosphate pathway, purine metabolism, and glycolysis/gluconeogenesis pathway.

**Fig 1:**
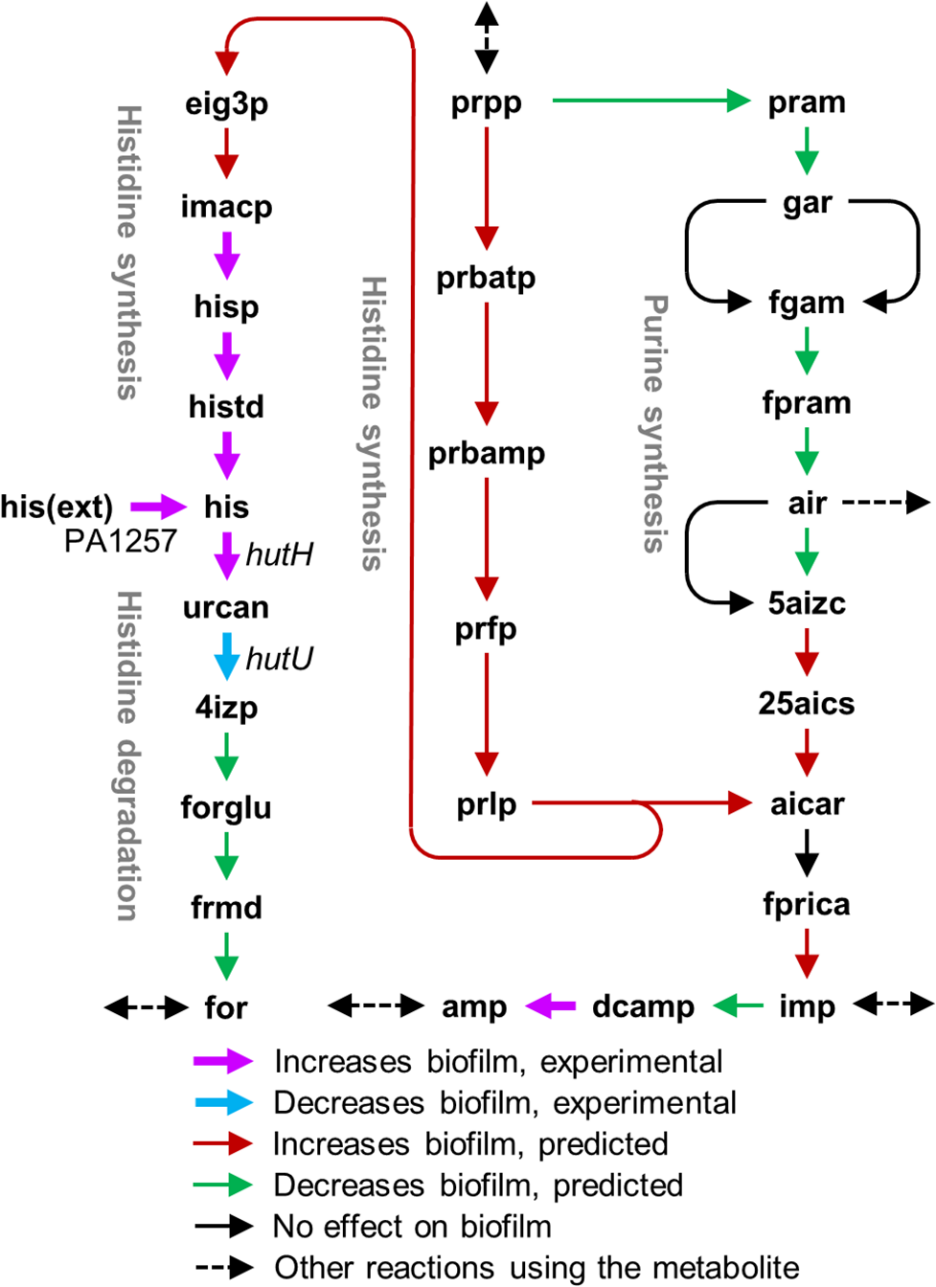
Alteration of biofilm formation by different reaction inhibitions in the histidine and purine synthesis pathways. Reactions indicated with magenta and cyan arrows were experimentally identified by Musken et al. [22]. We predicted reactions indicated with red and green arrows. Abbreviations: aicar, 5-phosphoribosyl-4-carbamoyl-5-aminoimidazole; prpp, 5-phosphoribosyl diphosphate; amp, adenylate. See S1 Supporting Information for the definition of the remaining of the abbreviations.

Notably, the fatty acid biosynthesis pathway, cysteine and methionine metabolism, and purine metabolism had more than five reactions whose inhibition decreased biofilm formation and more than five reactions whose inhibition increased biofilm formation. This may explain why knocking out the wrong component in a pathway may cause unexpected results even when the pathway itself is obviously directly related to a given phenotype. For example, *P*. *aeruginosa* can synthesize the purine precursor 5-phosphoribosyl-4-carbamoyl-5-aminoimidazole (aicar) from 5-phosphoribosyl diphosphate by two alternative routes in the purine and histidine synthesis pathways. We predicted that blocking the reactions in the histidine synthesis pathway would increase biofilm formation, whereas inhibition of five and three reactions in the purine synthesis pathway would decrease and increase biofilm formation, respectively (Fig 1). In addition, of the four reactions involved in converting 5-phosphoribosyl-4-carbamoyl-5-aminoimidazole (aicar) to adenylate (amp), we predicted that one would have no effect, one would increase, and one would decrease biofilm formation. The fourth reaction was associated with the gene PA3516, whose mutation is known to increase biofilm formation [22]. Different effects for different targets in the same pathway have also been experimentally observed. For instance, Musken et al. [22] identified that a mutation of *hutU*, which catalyzes the second step in the histidine degradation pathway, decreased biofilm formation. Thus, one would expect that blocking histidine uptake (PA1257) or the first step of the histidine degradation pathway (*hutH*) would also decrease biofilm formation; however, they found the opposite effect for these mutants (Fig 1).

The number of putative target reactions that reduce biofilm formation was relatively large with 78 experimentally determined (S3 Table) and 126 predicted (S7 Table) targets. We further reduced this set by filtering out reactions that were associated with genes whose mutations were known to increase the tolerance to at least one antimicrobial or have human homologs (see Materials and Methods). This resulted in a set of 56 putative target reactions against *P*. *aeruginosa* biofilm (S8 Table), 33 of which were predicted from our metabolic network analysis. Two of these reactions were associated with reduced tolerance mutants to at least one antimicrobial and 11 reactions were associated with attenuated virulence mutants, making these targets attractive from a drug design perspective (Table 1). Note that we predicted target reactions that would reduce biofilm formation and antimicrobial tolerance, properties that could not be determined from the experimentally identified mutants alone. However, we did not identify any target reaction that would simultaneously reduce biofilm formation, antimicrobial tolerance, and virulence.

**Table 1:** Putative target reactions against biofilm that also would decrease antimicrobial tolerance or attenuate virulence.

Reaction name	Reaction	Genes	Identification method^a^
*Reduced antimicrobial tolerance*			
N-acetyl-g-glutamyl-phosphate reductase	acg5sa + nadp + pi < = > acg5p + h + nadph	*argC*	P
Formimidoylglutamase	forglu + h2o = > frmd + glu-L	*hutG* or PA3175	P
*Attenuated virulence*			
1,2-diacyl-sn-glycerol 3-phosphate synthesis	1.02 2tocdACP + glyc3p + 0.26 hdeACP + 0.06 ocdACP + 0.66 palmACP = > 12dag3p_PA + 2 ACP	(*plsY* or *plsX* or *plsB*) and (*lptA* or *olsA*)	E
Isochorismate Synthase	chor = > ichor	*pchA*	E
Orotidine-5-phosphate decarboxylase	h + orot5p = > co2 + ump	*pyrF*	E
Protoporphyrinogen oxidase	1.5 o2 + pppg9 = > 3 h2o + ppp9	*hemY* or *hemK*	E
Arginine N-succinyltransferase	arg-L + succoa = > sucarg + coa + h	*aruF* and *aruG*	E
1-hydroxyphenazine synthase	h + nadh + o2 + pca = > 1hphe + co2 + h2o + nad	*phzS*	P
Phenazine-1-carboxylic acid synthesis, step 1	chor + gln-L = > a4dic + glu-L	*phzE1* or *phzE2*	P
Phenazine-1-carboxylic acid synthesis, step 2	a4dic + h2o = > dhha + h + pyr	*phzD1* or *phzD2*	P
Rhamnosyltransferase chain A	3hdeACP + coa = > 3hdccoa + ACP	*rhlA*	P
Isochorismate-pyruvate lyase	ichor = > sal + pyr	*pchB*	P
Pyochelin synthesis *pchDG*	salamp + cysamp = > hpthiazoline + 2 amp + h2o + h	*pchG* and *pchD*	P

^a^ E, reaction associated with an experimentally determined mutant; P, predicted.

An extension of the present analysis would be to predict pairs or higher-order combinations of reactions that simultaneously, but not separately, reduce biofilm formation. This would allow us to probe potential redundancies in the cellular metabolism that relates to biofilm formation. Computationally, this has been addressed using constraint-based methods to predict synthetically lethal double deletion mutants [28, 29]. Performing a similar analysis using the proposed kinetic models would require developing efficient methods to probe a large multidimensional space, given the computational burden of evaluating the kinetic model. A potential solution could be to use a constraint-based approach to create a list of promising pairs, triplets, etc. of reactions and, then, use the kinetic model to refine the list.

### Predicted metabolic differences between biofilm and planktonic phenotypes

We investigated the metabolic flux and metabolite concentration differences between stationary and biofilm cultures of *P*. *aeruginosa* to increase the knowledge base of biofilm-specific physiology. In particular, we focused on the experimental study carried out by Costaglioli et al. [11], in which they analyzed gene expression profiles of planktonic and biofilm cultures under similar conditions. We considered a metabolic flux or metabolite concentration to be substantially different between the biofilm and planktonic cultures if its median change was larger than two-fold, and if all the changes occurred in the same direction in all simulations using the ensemble of reference flux distributions. Based on the simulation results, we predicted that 15 reactions had a considerable flux increase and nine reactions had a considerable flux decrease in the biofilm conditions (Table 2). The increased-flux reactions were mainly involved in the anthranilate degradation pathway, the pyochelin synthesis pathway, and the Psl synthesis pathway, causing substantial increase in the flux through these pathways. In contrast, of the nine decreased-flux reactions, only the two reactions associated with the genes *pqsABCD* and *pqsH* appeared to have a functional effect (i.e., a reduced synthesis of PQS). The decreases on the other reactions were compensated by increases in other neighboring reactions. We can derive several insights from these predictions. For instance, the predicted increased production of the low affinity siderophore pyochelin, and the fact that the genes involved in the synthesis of the high affinity siderophore pyoverdine were not differentially expressed, suggest that the biofilm experienced a moderate iron starvation even though it was grown in the same medium as the planktonic culture.

**Table 2:** Reactions with predicted median flux ratio change of at least 2-fold in biofilm cultures compared to stationary cultures.

Reaction name	Reaction	Genes	GE ratio^a^	Flux ratio^b^		
				Median	Min.	Max.
*Down-regulated*						
**HHQ synthesis** ^c^	**3oxdeACP + anth + h = > ACP + co2 + h2o + hhq**	***pqsABCD***	**0.60**	**0.47**	**0.33**	**0.79**
**Probable FAD-dependent monooxygenase (PQS synthesis)** ^c^	**fadh2 + hhq + o2 = > fad + h2o + pqs**	***pqsH***	**0.99**	**0.47**	**0.33**	**0.79**
Acyl-ACP:malonyl-ACP C-acyltransferase (decarboxylating)	acACP + h + malACP = > ACP + actACP + co2	*fabB* or *fabF1* or PA5174	0.75	0.44	0.25	0.77
Glutamine transaminase	glu-L + pydx5p = > akg + pyam5p	*glyA1* or *glyA2*	0.22	0.18	0.15	0.34
Alanine transaminase	ala-L + pydx5p = > pyam5p + pyr	*glyA1* or *glyA2* or *glyA3*	0.37	0.29	0.24	0.57
3-deoxy-D-manno-octulosonic acid transferase	PA_lipidA + ckdo = > PA_KDOlipidA + cmp + h	*waaA*	1.13	0.23	0.08	0.74
Phosphate transport via ABC system	atp + h2o + pi[e] = > adp + h + 2 pi	*pstABCS*	0.24	0.27	0.25	0.34
L-serine deaminase	ser-L < = > nh4 + pyr	*sdaAB*	0.45	0.28	0.09	0.43
O-succinylhomoserine lyase (H2S)	h2s + suchms = > hcys-L + succ	*metZ*	0.58	0.44	0.35	0.57
*Up-regulated*						
**Psl synthesis** ^c^	**3 gdpman + udpg + dtdp6dm = > psl[e] + 3 gdp + udp + dtdp**	***pslACDEFGHIJKL***	**0.88**	**2.87**	**1.22**	**6.86**
Anthranilate 1,2-dioxygenase	anth + nadph + 2 h + o2 = > catechol + co2 + nadp + nh3	*antA*	5.64	2.48	1.90	6.01
3-oxoadipyl-CoA thiolase	coa + oxadpcoa = > accoa + succoa	*pcaF*	1.93	2.06	1.32	3.24
Catechol 1,2-dioxygenase	catechol + o2 = > muc	*catA*	4.30	2.48	1.90	6.01
3-oxoadipate enol-lactonase	5odhf2a + h2o < = > 3oxadp + h	*pcaD*	3.16	2.06	1.32	3.24
3-oxoadipate CoA-transferase	3oxadp + succoa < = > oxadpcoa + succ	*dhcAB*	0.92	2.06	1.32	3.24
NAD(P) transhydrogenase	2 h[e] + nadh + nadp < = > 2 h + nad + nadph	*pntAA* and *pntB*	1.12	3.51	1.99	4.88
Isochorismate synthase	chor = > ichor	*pchA*	6.24	3.91	3.46	4.22
Isochorismate-pyruvate lyase	ichor = > sal + pyr	*pchB*	7.86	3.91	3.46	4.22
Pyochelin synthesis *pchE*	cys-L + atp = > cysamp + ppi	*pchE*	8.97	3.91	3.46	4.22
Pyochelin synthesis *pchD*	sal + atp + h = > salamp + ppi	*pchD*	3.30	3.91	3.46	4.22
Pyochelin synthesis *pchDG*	salamp + cysamp = > hpthiazoline + 2 amp + h2o + h	*pchDG*	2.73	3.91	3.46	4.22
Pyochelin synthesis *pchEF*	hpthiazoline + cysamp = > hpbthiazoline + amp + h2o + h	*pchEF*	3.86	3.91	3.46	4.22
Pyochelin synthesis *pchGF*	hpbthiazoline + nadph + h = > dmpyochelin + nadp	*pchGF*	1.94	3.91	3.46	4.22
Pyochelin synthesis *pchF*	dmpyochelin + amet + h2o = > pyochelin + ahcys + 3 h	*pchF*	1.66	3.91	3.46	4.22

^a^ GE ratio, gene expression ratio between biofilm and stationary planktonic conditions.

^b^ The median, minimum (Min.), and maximum (Max.) values of the flux ratio of each reaction in the set of simulations carried out using each member of the ensemble of flux distributions.

^c^ Biofilm-related reaction with considerable flux change but no significant gene expression changes.

**Fig 2:**
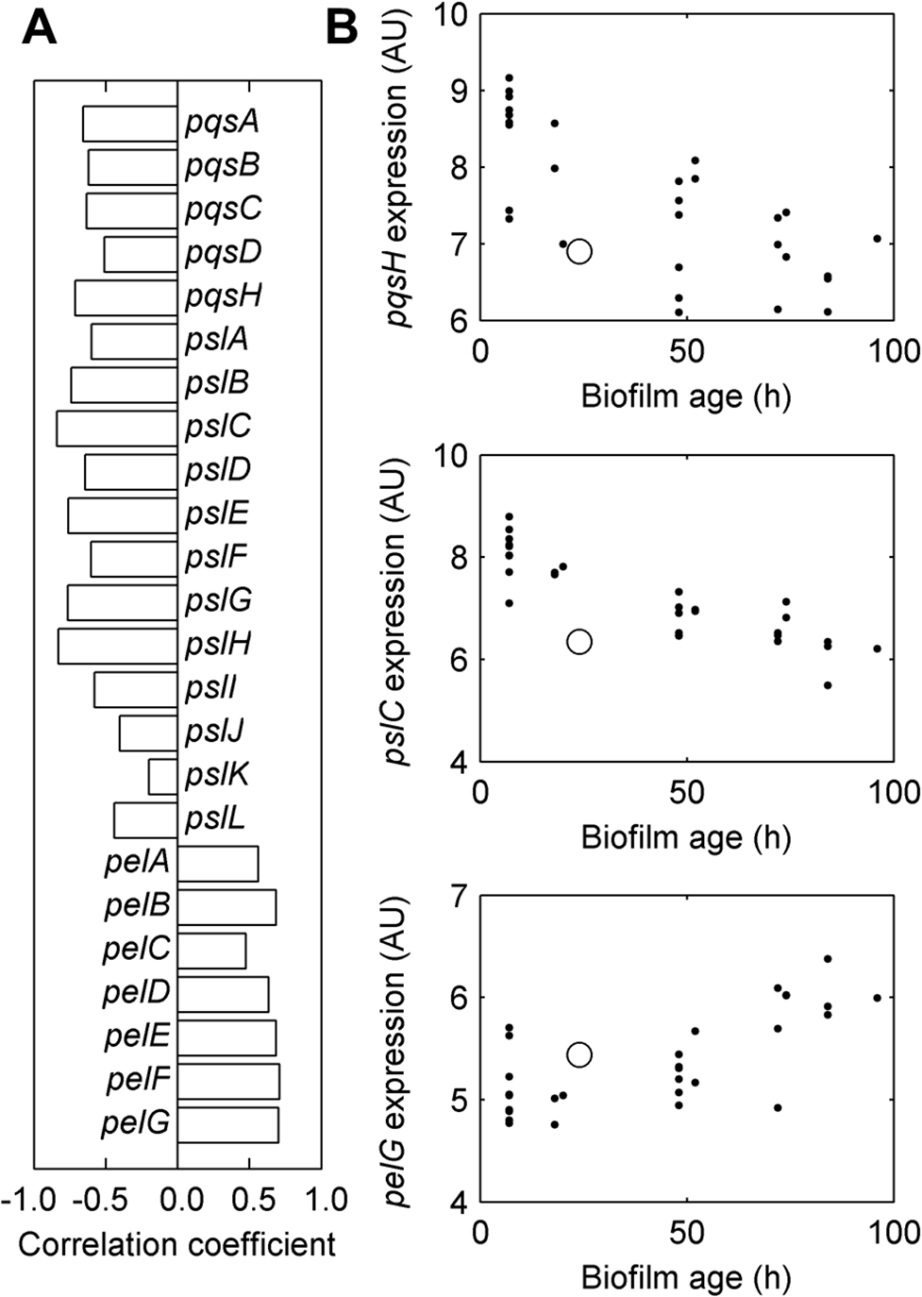
Expression of *pqs*, *psl*, and *pel* operons as a function of biofilm age. (A) Correlation coefficient of the expression intensity of the genes in the *pqs*, *psl*, and *pel* operons and biofilm age (h). (B) Expression of one gene from each operon as a function of biofilm age. Gene expressions are shown in log_2_ scale. Each dot corresponds to one condition in the dataset of gene expression for *P*. *aeruginosa* PAO1 biofilms. The circles correspond to the data obtained by Costaglioli et al. [11]. AU, arbitrary units; h, hours.

We predicted that the synthesis of two biofilm-related molecules would have a considerable flux change without significant expression changes of the genes associated with the corresponding enzymatic reactions. The production of PQS was reduced, while the synthesis of the polysaccharide Psl was increased. PQS is a quorum-sensing signal that regulates the production of virulence factors and biofilm development in a complex regulatory network with the N-acylhomoserine lactone-dependent quorum-sensing systems *las* and *rhl* [30]. Diggle et al. [31] reported increased biofilm formation in *P*. *aeruginosa* cultures treated with exogenous PQS after 72 h. Similarly, Guo et al. [32] reported that PQS promoted biofilm formation, but in their experiments the enhancement occurred within the first hour of incubation, and the cultures with and without exogenous PQS had similar biofilm formation rates from 1 to 24 h. This observation, coupled with the observation that the genes involved in PQS synthesis were not up-regulated in the biofilm, suggests that PQS may be more important at the early stages of biofilm formation and that its synthesis is down-regulated as the biofilm matures. Moreover, our simulation results indicated that further repression of PQS synthesis was achieved by increasing anthranilate degradation. We investigated whether PQS is indeed down-regulated as the biofilm matures, rather than as a consequence of the specific experimental condition in the study by Costaglioli et al. [11]. To this end, we analyzed the expression of the PQS synthesis genes (*pqsABCDH*) in all datasets available in the Gene Expression Omnibus (GEO) database [33] for the wild-type PAO1 strain of *P*. *aeruginosa* growing in biofilm (see Materials and Methods for details on processing the gene expression data). We found that there was a significant negative correlation between the expression of the PQS synthesis genes and the biofilm age (Fig 2).

**Fig 3:**
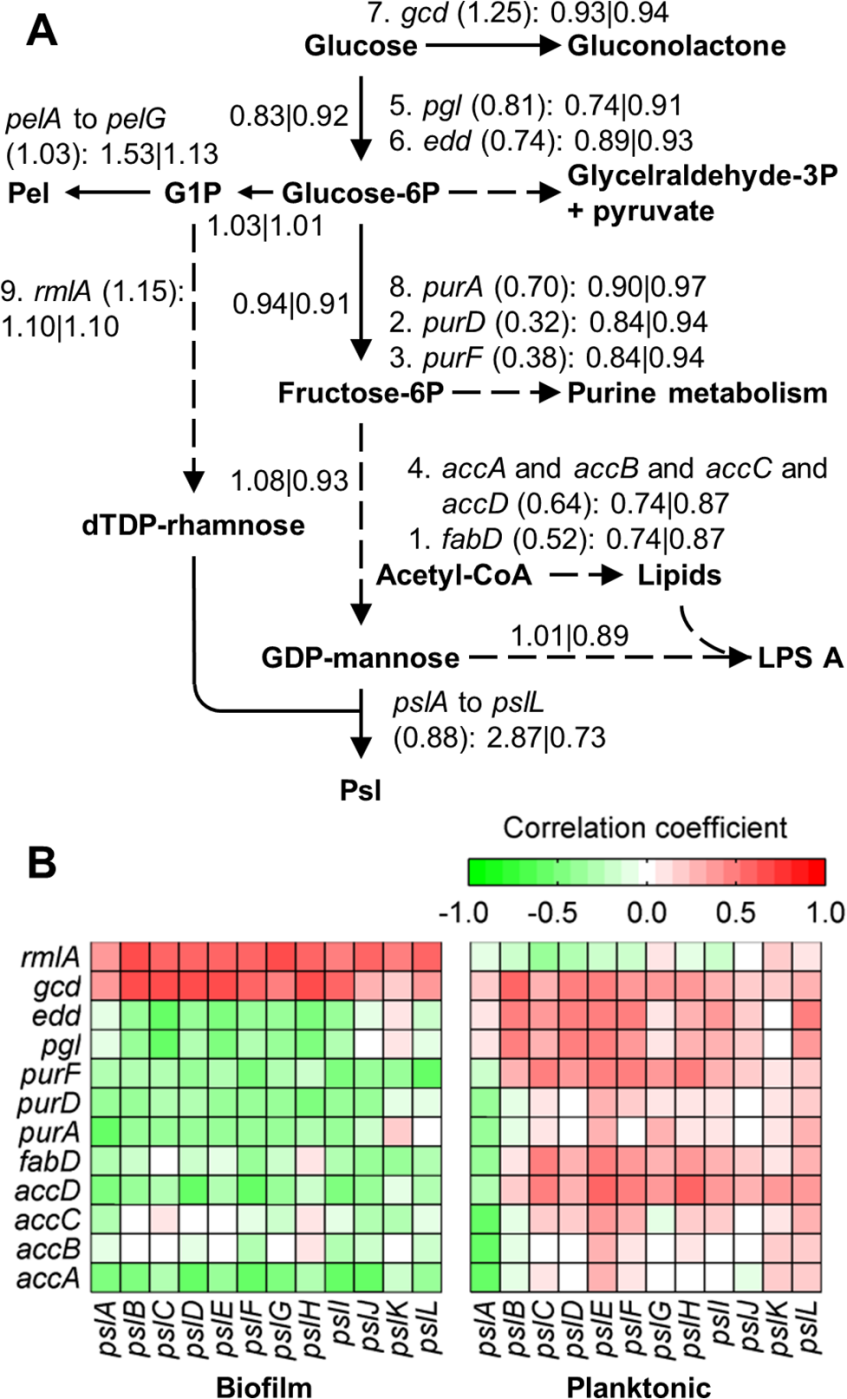
Coordinated regulation of Psl synthesis. (A) Sketch of the metabolic pathways involved in the increase of Psl and Pel production rates in our simulations. The figure shows the genes associated with the nine reactions that had the highest effect on Psl production. The number to the left of each gene name denotes the rank of the corresponding reaction. The number in parentheses denotes the overall gene expression ratio between the biofilm and the stationary cultures. The numbers to the left and right of the vertical bar denote the median flux ratios of the reactions associated with the genes in simulation with or without the gene expression ratios of the genes *accABCD*, *fabD*, *purADF*, *pgl*, *edd*, *gcd*, and *rmlA*. Only *psl*, *pel*, and those genes whose regulation contributes to increasing Psl and Pel production are shown. (B) Correlation between the expression of the genes that contribute to increasing Psl production and the *psl* operon genes for biofilm and stationary cultures. Solid and dashed arrows indicate single and multiple reaction steps in the model, respectively.

Fig 2 also shows that the expression of the genes involved in the synthesis of Psl (*psl* genes) and Pel (*pel* genes), the two major exopolysaccharides forming the extracellular matrix in biofilms of the PAO1 strain [34], was correlated with the biofilm age, although with opposing signs. This is consistent with the observation that *P*. *aeruginosa* strains produce predominantly one polysaccharide at any given time [35]. But unexpectedly, the *psl* and *pel* genes were not up-regulated in the biofilm compared with the stationary culture in the study by Costaglioli et al. [11]. A possible explanation for this observation could be that most of the nutrients in the medium have been consumed after 24 h and the synthesis of exopolysaccharides has been stalled in both cultures. Nonetheless, our simulations predicted that there was an increase in the synthesis rate of Psl (median flux ratio = 2.87, minimum flux ratio = 1.22) in the biofilm with respect to the stationary culture. There was also an increase, although small, in the Pel synthesis (median flux ratio = 1.53, minimum flux ratio = 1.32). To understand what caused this result in our simulations, we evaluated the effect of the overall gene expression change of each reaction on the predicted Psl synthesis rate (see Materials and Methods). We found that the increase in Psl synthesis was mainly caused by the down-regulation of a few reactions in pathways competing for Psl precursors. Fig 3A shows a sketch of the pathways associated with the increase in the synthesis of Psl. This figure also shows the genes associated with the nine reactions that had the highest effect on Psl production (the addition of more reactions did not have a significant effect), as well as the predicted median flux ratios in simulations with or without the gene expression changes of all of these genes. Psl production was increased by down-regulating the genes of seven of these reactions (although only two of them, *purD* and *purF*, by more than two-fold) and the other two reactions had a gene expression ratio only slightly higher than 1.0 (*gcd* and *rmlA*). Interestingly, the sensitivity of the nine reactions coincided with the correlation between their gene expression and the expression of the Psl pathway genes in the datasets obtained from the GEO database (Fig 3B). In other words, the gene expression of the reactions whose down-regulation increased Psl synthesis had a negative correlation with the gene expression of the Psl pathway, whereas the reactions whose up-regulation increased Psl synthesis had a positive correlation. Notably, with the exception of *gcd* (PA2290), the opposite was observed in planktonic cultures: the gene expression of the reactions whose down-regulation increased Psl synthesis had a positive correlation with the gene expression of the Psl pathway, whereas the reactions whose up-regulation increased Psl synthesis had a negative correlation (Fig 3B). This result led to the hypothesis that *P*. *aeruginosa* may increase the production of Psl (and possibly Pel) by not only regulating the genes in their synthesis pathways, but by down-regulating pathways competing for Psl precursors in a biofilm-specific manner.

Importantly, the kinetic model-based analysis allowed us to make an enhanced interpretation of the gene expression data. Inspection of the gene expression data indicates that 18 of the genes in the metabolic network were up-regulated and 30 down-regulated in the biofilm culture (S9 Table). One would infer that the 20 reactions associated with the up-regulated genes have an increased flux, and the 36 reactions associated with the down-regulated genes have a decreased flux. However, only nine and four of these reactions had a considerable flux increase or decrease in our simulations, respectively (reactions with a gene expression ratio greater than 2.0 or lower than 0.5 in Table 2). Table 2 also shows that six increased-flux and five decreased-flux reactions had an overall gene expression change less than two-fold. Thus, these reactions would not have been identified based on gene expression analysis alone. Conversely, there were four and eight reactions with overall gene expression ratios larger than 2.0 and smaller than 0.5, respectively, but for which the predicted flux changes were moderate (S10 Table). We expect that the predicted flux changes represent a better picture of the true metabolic changes than the conclusions that one can draw using only the gene expression data. This claim is supported by previous studies that demonstrated that metabolic network models combined with gene expression data are better predictors of metabolic flux changes than gene expression data alone [17, 36].

A critical aspect of biofilms that we have not explicitly accounted for is the progression of biofilm formation. This analysis would require a consistent set of gene expression data at different time points and measurements of metabolite concentration at the initial condition (i.e., the reference condition). An alternative approach would be to examine the dynamics of biofilm formation using aggregated time-dependent and averaged expression data from the datasets available in the GEO database. We could then employ our modeling framework, under pseudo-steady-state approximation, for each time point and construct a time-dependent solution. However, there are a number of assumptions and technical difficulties that would need to be resolved before such a technique would be considered reliable enough to infer biofilm dynamics. For instance, the gene expression data from the GEO database were collected under different conditions related to time, temperature, medium, culture mode, chemical treatments, etc. All of these conditions will, to some extent, influence the details of the biofilm formation analysis.

### Dysregulation of secondary metabolites rather than energy shortage may explain low biofilm formation in anthranilate degradation mutants

Costaglioli et al. reported that several genes in the anthranilate degradation pathway of *P*. *aeruginosa* were up-regulated in biofilms compared to stationary cultures, and that the mutation of such genes led to decreased biofilm formation [11]. They suggested that the decrease in biofilm formation is caused by the inability of the mutants to use anthranilate as an energy source. However, simulations inhibiting anthranilate degradation reactions predicted no decrease in biomass growth rate (minimum relative growth rate = 1.0) or ATP concentration (minimum relative ATP concentration = 0.94). Similarly, FBA simulations predicted that blocking anthranilate degradation affects the optimal biomass growth rate only when tryptophan is the only carbon source, a scenario that is unlikely to occur, because the biofilm cultures were grown in a rich medium. Moreover, Brandenburg et al. [37] recently reported that tryptophan inhibits *P*. *aeruginosa* biofilm formation. Thus, this suggests that the mechanism for biofilm reduction is not directly related to a reduction of energy production.

**Fig 4:**
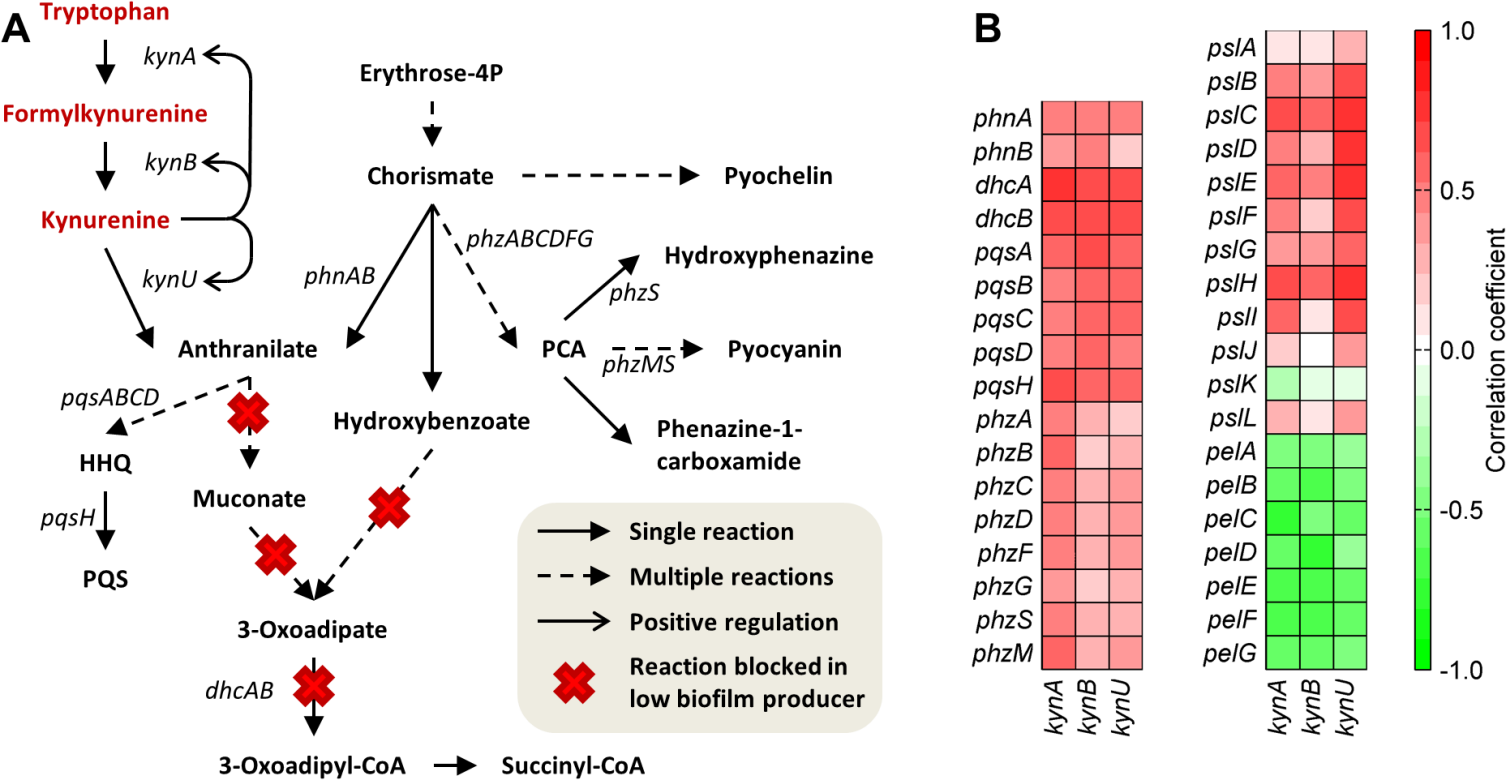
Dysregulation of secondary metabolites related to biofilm formation by inhibition of anthranilate degradation. (A) Sketch of the metabolic pathways involved in anthranilate and chorismate metabolism. Metabolite names written in red were predicted to increase when the reactions marked with a red x, which correspond to the low biofilm producers identified by Costaglioli et al. [11], were inhibited. (B) Correlation of the gene expression intensity of genes associated with anthranilate- and chorismate-derived secondary metabolites, *psl* and *pel* genes, with the genes of the kynurenine pathway.

Anthranilate is a key node in a complex pathway for the synthesis of several important secondary metabolites for *P*. *aeruginosa* physiology derived from chorismate and tryptophan (Fig 4A). In simulations of the mutants that reduced biofilm formation in the study by Costaglioli et al. [11], concentrations of 19 metabolites changed by a factor of two or more in at least one mutant (S11 Table). Of the altered metabolites, only tryptophan, formylkynurenine, and kynurenine appeared in the set of metabolites with increased concentration in the low biofilm producers (these mutants were used in the computation of the sets of metabolites associated with the biofilm-reducing and biofilm-increasing reactions). Formylkynurenine and kynurenine are intermediate metabolites in the conversion of tryptophan to anthranilate through the kynurenine pathway, and kynurenine is known to be an inducer of the genes (*kynABU*) in this pathway [38]. Notably, the expression of *kynABU* correlated with the expression of genes downstream of anthranilate and chorismate, as well as with *psl* genes, but had a negative correlation with the *pel* genes in the dataset for *P*. *aeruginosa* biofilms (Fig 4B). Thus, we hypothesized that kynurenine accumulation led to up-regulation of *kynABU* and increased flux to anthranilate, which could not be degraded and, therefore, it was diverted to the synthesis of other metabolites, such as PQS, pyocyanine, and hydroxyphenazine, altering the normal development of the biofilm. This hypothesis is supported in part by a study by Oglesby et al. [39], who demonstrated the link between the regulation of anthranilate degradation genes (*antABC*) and the synthesis of PQS by showing that the expression of *antABC* affected the production of PQS, and that overexpression of *pqsR* (a quorum-sensing regulator co-induced by PQS) inhibited *antABC* expression.

### Conclusions

We have investigated the metabolism of *P*. *aeruginosa* under biofilm conditions using a genome-scale kinetic model and gene expression profiles. Specifically, we identify potential target reactions to reduce biofilm formation using metabolite concentration changes predicted to be specific for the inhibition of either biofilm-reducing or biofilm-increasing reactions, in contrast with previous modeling analysis that used biomass growth as a surrogate of biofilm formation and failed to find biofilm-specific essential genes. We also predicted the metabolic differences between biofilm and stationary cultures and hypothesized a mechanism for regulating the synthesis of important biofilm-related molecules, such as Psl and PQS. Finally, we proposed a mechanistic explanation of why mutants defective in anthranilate degradation had a reduced biofilm formation. Our simulation data showed that the predicted mechanisms for Psl synthesis regulation and the effect of blocking anthranilate on biofilm formation had a subtle connection with the co-expression of several of the involved genes. Thus, our work highlighted how metabolic network analysis could generate non-intuitive hypotheses regarding poorly understood mechanisms of biofilm development.

## Materials and Methods

### Metabolic network of *P*. *aeruginosa*

We constructed a kinetic model based on the metabolic network reconstruction of Oberhardt et al. [18], with a number of modifications, as detailed in S1 Supporting Information. In particular, we completed the anthranilate degradation and pyochelin synthesis, and modified the lipopolysaccharide synthesis pathways, because these pathways are associated with genes differentially expressed in biofilm cultures in the data from Costaglioli et al. [11]. We also included the synthesis pathways for the major exopolysaccharides produced by the strain PAO1, Psl and Pel [34]. For the simulated growth conditions, the modified model had 685 reactions associated with 876 genes and 504 metabolites.

### Kinetic model

We used our previously developed modeling framework [17] to construct a genome-scale kinetic model for the metabolic network of *P*. *aeruginosa*. Briefly, the constructed model can be used to simulate a specific condition or to predict the metabolic fluxes and metabolite concentration changes brought about by gene expression changes between two conditions: a reference condition and the condition of interest. Assuming pseudo-steady-state conditions, the kinetic model can be represented as

S⋅r(v,g,c)=0, (1)

where **S** denotes the stoichiometric matrix of the metabolic network reconstruction, **r** represents the vector of reaction rates, **v** denotes the reference flux distribution, **g** represents the vector of gene expression ratios between the condition of interest and the reference condition, and **c** represents the vector of normalized metabolite concentrations. Thus, the model requires two sets of parameters, a flux distribution for one of the conditions and the gene expression ratios between the two conditions (see below). For the kinetic expression, we used a particular case of generalized mass action kinetics [40]. For a general irreversible reaction

$$\sum_{i}a_{i}A_{i}\to \sum_{j}b_{j}B_{j},$$ (2)

we used the expression form

$$r=vg\left(\frac{\prod_{i}{\left[A_{i}\right]}^{a_{i}}}{\prod_{j}{\left[B_{j}\right]}^{b_{j}}}\right)$$ (3)

where *a*_*i*_ and *b*_*j*_ denote the stoichiometric coefficients of species *A*_*i*_ and *B*_*j*_, respectively, *r* represents the reaction rate, the parameter *v* denotes the value of the reaction rate or flux through the reaction at a reference condition, *g* represents the overall gene expression ratio (between a condition of interest and a reference condition) of the genes associated with the reaction, and the square brackets denote normalized metabolite concentrations. Note that we made the rate of irreversible reactions a function of their products to allow metabolites downstream of an irreversible reaction to have an effect on the flux through a pathway. In a living cell, these reactions are under control of multiple biological and regulatory processes not included in the model. The proposed kinetic expressions partially allow us to capture these processes; otherwise, the flux through a pathway would depend only on the substrates and enzyme level of its *first* irreversible reaction. We assumed that this scenario is not likely to occur in metabolic networks because enzyme level changes of downstream reactions would then not affect the flux through the pathway. In fact, gene expression data clearly show differential gene expression regulation of downstream reactions.

Similarly, for a general reversible reaction, we used the expression form

$$r=g\left(v^{f}\prod_{i}{\left[A_{i}\right]}^{a_{i}}-v^{b}\prod_{j}{\left[B_{j}\right]}^{b_{j}}\right),$$ (4)

where *v* ^*f*^ and *v* ^*b*^ were determined as follows:

$$v^{f}=\beta v\;\text{and}\;v^{b}=(\beta -1)v,\;\text{if}\;v>0,$$ (5)

$$v^{f}=(\beta -1)v\;\text{and}\;v^{b}=\beta v,\;\text{if}\;v<0,$$ (6)

where *β* is a parameter that relates the rate of the forward and backward reactions to the overall flux at the reference condition. The value of *β* depends on the equilibrium constant and on the reactant’s concentration at the reference condition (see S1 Text). However, these data are generally not available as in the experiments analyzed here. Therefore, assuming that the model behavior had a low sensitivity to individual *β*s, we took all *β*s to have the same value, except for reactions in parallel pathways that must satisfy additional thermodynamic constraints as detailed in Vital-Lopez et al. [17]. The lower limit of *β* is 1.0. A value of *β* close to 1.0 corresponds to a reaction very far from the equilibrium, whereas a large value of *β* corresponds to near-equilibrium reactions. We assumed that all reversible reactions are near equilibrium and set *β* = 50 for reaction that do not participate in parallel pathways. The values of *β* for reactions in parallel pathways are provided in S2 Supporting Information. The simulation results had low sensitivity to *β* in the neighborhood of 50 (simulations with *β* = 30 or *β* = 100 produced similar results; see S12 Table). The simulations become more sensitive to *β* as the parameter approaches 1.0. Moreover, from practical experience, the model becomes unstable for values of *β* close to 1.0. For example, 70% of the simulations fail to stay at steady state when simulating the reference condition using *β* = 1.5. As we previously showed, approximating all *β*s to a single relatively large value produced satisfactory results [17]. More details of the modeling framework are provided in Vital-Lopez et al. [17].

Note that we could have included the concentration of products in the forward reactions and the concentration of substrates in the backward reactions for the reversible reactions. However, we decided not to include them because the simpler kinetic expressions already captured the effect of both substrates and products in the reaction rate.

In all analyses in this work, we simulated the model under the pseudo-steady-state assumption. Under this assumption, we have previously shown that the proposed modeling framework produced satisfactory results [17], even in the absence of alternative regulatory mechanisms and with the coarse approximation for some model parameters, such as the reference flux distribution and *β*. In fact, our modeling approach outperformed the method proposed by Moxley et al. [36], which incorporated known enzyme-metabolite interactions.

Thus, the proposed kinetic model has four sets of parameters, namely, the gene expression ratios **g**, the reference flux distribution **v**, the exponent of the concentrations *a*_*i*_ and *b*_*j*_, and the *β*s. In this work, the gene expression ratios were directly computed from gene expression data. We used an ensemble of random reference flux distributions, instead of a single flux distribution, to account for the lack of data to estimate an accurate flux distribution. The exponents of the concentrations are stoichiometric coefficients of the metabolites of each reaction and are directly taken from metabolic network model. As we mention above, with exception of the reactions that participate in parallel pathways, we approximate all *β*s to a single value without considerably affecting the simulation results. We provided all the parameter sets, including the *β*s for reactions in parallel pathways, in S2 Supporting Information.

### Gene expression data

We used the gene expression data obtained by Costaglioli et al. [11] to simulate the metabolic changes in their experiments. We obtained the raw data with accession number GSE30021 from the GEO database [33] and processed the data using the MATLAB (2011b, The MathWorks Inc., Natick, MA) function affygcrma. We further smoothed the processed data to reduced non-random systematic error. Briefly, for each microarray in the dataset, we computed a correction factor for each gene such that the systematic deviations in the signal intensity of each microarray with respect to the average signal intensity of the dataset were reduced. The correction factor is equivalent to what is obtained using the MATLAB function malowess, although we used an in-house-developed function (S1 Fig). For the analysis of gene expression of multiple datasets of biofilm and planktonic cultures, we looked for microarray data in the GEO database using the search term “Pseudomonas aeruginosa PAO1 [porgn:__txid208964].” From the search results, we selected the microarray data that were associated with the wild-type strain (e.g., no mutants), where cells were grown in biofilms or liquid planktonic cultures, and the raw data (i.e., CEL files) were available. In addition, we included other datasets from the literature that met the conditions mentioned above, but were not retrieved from the search results (see S13 Table for the full list of the microarray data used). We processed these datasets the same way as described above.

### Reference flux distribution

Experimental measurements to determine the reference flux distribution for the exponential, stationary, or biofilm cultures in the study by Costaglioli et al. [11] were not available. Therefore, we randomly sampled the space of feasible flux distributions to generate ensembles of reference flux distributions, and then carried out the simulations on each member of the ensemble. Briefly, we created two ensembles of 100 random reference flux distributions each for the exponential and stationary cultures. First, we carried out a flux variability analysis to compute the lower and upper bounds for each reaction. Then, we generated random flux vectors that were contained within these bounds using Latin hypercube sampling [41]. Finally, for each random flux vector, we computed a flux distribution that was as close as possible to the random flux vector while satisfying the mass balance, thermodynamics, simulation condition constrains (see S1 Text for details). For the exponential cultures, we assumed that the reference flux distributions could have any uptake rates for the nutrients in the Luria-Bertani medium and oxygen, as long as the biomass yield was at least 50% of the optimal biomass yield. We computed optimal biomass yield for exponential cultures by minimizing the total carbon uptake for a fixed biomass growth rate, without any restrictions in nutrients or oxygen uptake rates. For the stationary cultures, the reference flux distributions were also constrained to have a biomass yield of at least 50% of the optimal yield under the same conditions. We computed the optimal biomass yield for stationary cultures in the same way that the exponential cultures, but with two additional constrains. We restricted the oxygen consumption to 20% of the optimal uptake rate for the exponential culture and the major carbon source to contribute no more than 25% of the total used carbon (assuming that the preferred carbon source would be mainly used during the exponential growth-phase).

### Prediction of essential reactions using FBA

We predicted the effect of completely blocking each reaction on the biomass growth rate by solving the following FBA problem:

$$\begin{array}{l} \underset{y}{\text{max}}\;y_{bio}\\ \text{s.t.}\\ S\cdot y=0\\ y\geq l\\ y\leq u\\ y_{i}=0, \end{array}$$ (7)

where **S** denotes the stoichiometric matrix of the metabolic network reconstruction, **y** represents the vector of fluxes, *y*_*bio*_ and *y*_*i*_ denote the biomass growth and the flux of the *i*-th reaction, respectively, and **l** and **u** represent the vectors of the lower and upper flux bounds, respectively. The lower and upper bounds are provided in the S2 Supporting Information. Note that we kept the biomass composition constant in the FBA, whereas we allowed variations in the kinetic model simulations. If constraining the flux of the *i*-th reaction to zero blocked the biomass growth, then the reaction was considered FBA-predicted essential, otherwise, the reaction was considered FBA-predicted non-essential.

### Simulation of reaction inhibitions using the kinetic model

We simulated the effect of inhibiting a metabolic reaction under exponential and biofilm culture conditions. For both conditions, we obtained an ensemble of reference flux distributions as described above. We simulated reaction inhibitions by multiplying each reaction rate, one at a time, by a factor of 0.0 or 0.01 for non-essential and essential reactions, respectively. Then, we solved Eq 1 for each reaction inhibition and each member of the ensemble of reference flux distributions. Note that for the simulations of the exponential cultures, we used the exponential cultures as the reference conditions themselves and, therefore, the simulations did not require gene expression (i.e., **g** = 1 in Eq 1). For the biofilm cultures, we carried out the simulations using the same reference condition as in the analysis of the metabolic differences between the biofilm and planktonic cultures. The reference condition was the stationary culture because this culture is physiologically closer to the biofilm culture than the exponential culture [10, 11]. Thus, in addition to the reference flux distribution, these simulations required the gene expression ratio between the biofilm and stationary cultures [11].

### Scoring function to predict the effect of metabolic reaction inhibitions on biofilm formation

**Fig 5:**
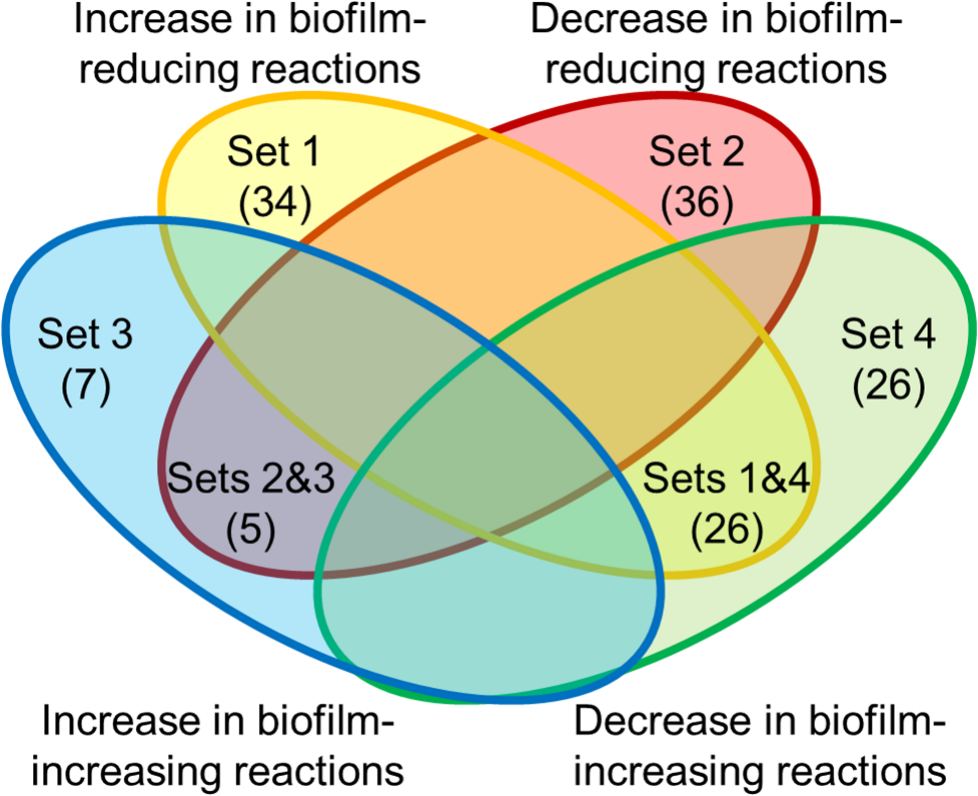
Definition of metabolite sets whose concentration changes were specific to either biofilm-reducing or biofilm-increasing reactions. The defined sets are as follows: set 1, metabolites that specifically increased when inhibiting biofilm-reducing reactions; set 2, metabolites that specifically decreased when inhibiting biofilm-reducing reactions; set 3, metabolites that specifically increased when inhibiting biofilm-increasing reactions; and set 4, metabolites that decreased when inhibiting biofilm-increasing reactions. Note that each metabolite set includes metabolites from two subsets of the Venn diagram. The number in parentheses indicates the number of metabolites in each subset.

For the inhibition of a metabolic reaction, we defined a scoring function to count how many metabolite concentration changes were in common with the concentration changes caused by inhibition of reactions associated with genes whose mutations are known to either reduce or increase biofilm formation [22] (see S3 Table for the list of the genes). We considered that a metabolite had a considerable concentration change if the median change was larger than two-fold and all the changes occurred in the same direction across the ensemble of reference flux distributions. Based on the predicted metabolite concentration changes caused by the inhibition of the biofilm-reducing and biofilm-increasing reactions, we defined four metabolite sets as follows (see Fig 5):

1. Metabolites whose concentrations increased when inhibiting at least one biofilm-reducing reaction (but their concentration did not decrease nor increase when inhibiting any biofilm-reducing or biofilm-increasing reactions, respectively).
2. Metabolites whose concentrations decreased when inhibiting at least one biofilm-reducing reaction (but their concentration did not increase nor decrease when inhibiting any biofilm-reducing or biofilm-increasing reactions, respectively).
3. Metabolites whose concentrations increased when inhibiting at least one biofilm-increasing reaction (but their concentration did not increase nor decrease when inhibiting any biofilm-reducing or biofilm-increasing reactions, respectively).
4. Metabolites whose concentrations decreased when inhibiting at least one biofilm-increasing reaction (but their concentration did not decrease nor increase when inhibiting any biofilm-reducing or biofilm-increasing reactions, respectively).

Then, for the inhibition of each reaction not used in the definition of any one of the metabolite sets, we defined the following scoring function:

$$s_{met,i}=-n_{1,i}-n_{2,i}+n_{3,i}+n_{4,i}$$ (8)

where *s*_*met*,*i*_ denotes the score for *i*-th reaction, and *n*_1,*i*_ to *n*_4,*i*_ denote the number of metabolites concentration changes caused by inhibiting the *i*-th reaction that were similar to the metabolite concentration changes of sets 1 to 4, respectively. Thus, we assumed that inhibition of reactions with a negative or a positive score are likely to reduce or increase biofilm formation, respectively.

### Filtering putative target reactions for biofilm reduction

We collected mutants with altered tolerance to at least one antimicrobial from eight different studies [42–49]. The studies covered 18 antimicrobials from 10 chemical classes and reported a combined total of 262 and 459 mutants with decreased and increased tolerance to at least one antimicrobial (S3 Supporting Information). We also collected mutants with attenuated virulence identified with a *Caenorhabditis elegans* model [50], a rat model of respiratory infection [51], and from the Virulence Factor Database (accessed October 20, 2014) [52]. We found 526 unique mutants with attenuated virulence (S3 Supporting Information). We used the Pseudomonas Genome Database [53] to obtain a list of *P*. *aeruginosa* genes with human homologs (S3 Supporting Information). We obtained sets of experimentally determined reactions associated with each phenotype (e.g., increased tolerance to at least one antimicrobial) based on the compiled lists of genes whose mutations altered a given phenotype and the gene-reaction relationships in the metabolic network model.

### Simulation of metabolic changes between biofilm and stationary planktonic cultures

We used the same simulation conditions to predict the metabolic differences between biofilm and stationary cultures as in the simulations of reaction inhibitions. That is, we used the same ensemble of reference flux distributions and the same gene expression data [11]. For each reference flux distribution in the ensemble, we computed a flux distribution and metabolite concentration changes for the biofilm culture by solving Eq 1.

### Simulation of the effect of gene expression changes on Psl synthesis

We carried out these simulations using the same ensemble of flux distributions and gene expression data as in the simulations to predict the metabolic differences between the biofilm and stationary cultures. We simulated the effect of the gene expression changes of a reaction by running the model (Eq 1) using all the gene expression changes except the gene expression changes for that reaction. We carried out this simulation for each reaction and determined which reaction had the largest effect on Psl synthesis (excluding the reaction for Psl synthesis).

## Supporting Information

S1 Supporting Information Metabolic network model of *P*. *aeruginosa*. An Excel file with the lists of the metabolic reactions and metabolites in the model.
(XLSX)

S2 Supporting Information Kinetic model of *P*. *aeruginosa* metabolic network. A compressed archive containing MATLAB files to simulate the metabolism of *P*. *aeruginosa* under biofilm conditions.
(ZIP)

S3 Supporting Information *P*. *aeruginosa* genes associated with altered drug sensitivity or virulence, and with human homologs. (XLSX)

S1 Text A text document that provides details on the estimation of reference flux distribution, reproducibility of the simulation results, model validation, alternative predictive functions of biofilm formation, validation of the scoring function, and definition of the parameter *β*. (DOCX)

S1 Table Essential reactions determined by FBA. (XLSX)

S2 Table Predicted reactions important for biomass growth for exponential and biofilm cultures. (XLSX)

S3 Table Biofilm-reducing and biofilm-increasing reactions of *P*. *aeruginosa* used to define metabolite sets indicative of biofilm effect. (XLSX)

S4 Table Genes found in the literature to affect biofilm formation. (XLSX)

S5 Table Metabolites that were altered specifically when inhibiting biofilm-reducing or biofilm-increasing reactions. (XLSX)

S6 Table Metabolic pathways with five or more metabolites in the metabolite sets specific for low and high biofilm producers. (XLSX)

S7 Table Reactions whose inhibition is predicted to alter biofilm formation. (XLSX)

S8 Table Reactions whose inhibition is predicted to alter biofilm formation after removing reactions associated with increased antimicrobial tolerance mutants and human homologs. (XLSX)

S9 Table Differentially expressed genes in biofilm with respect to stationary cultures in the study by Costaglioli et al. [11]. (XLSX)

S10 Table Reactions with differentially expressed genes that did not have a considerable flux change. (XLSX)

S11 Table Metabolites with a predicted concentration change larger than two-fold in at least one of the mutants that had a reduced biofilm formation in the study by Costaglioli et al. [11]. (XLSX)

S12 Table Effect of the parameter *β* on the predicted flux and metabolite concentration ratios. (XLSX)

S13 Table Microarray datasets used to determine gene expression correlations in biofilm and planktonic cultures. (XLSX)

S1 Fig Smoothing of the processed data for microarray GSM854799. (A) Smoothed and pre-smoothed data for the microarray with the highest deviation from the average expression intensity (GSM854799), compared to the average expression intensity of all microarrays in the dataset for *P*. *aeruginosa* PAO1 growing in biofilms (see S13 Table for microarray accession numbers). (B) Comparison of the microarray data smoothed using the in-house-developed smoothing function and the MATLAB function malowess.
(TIF)
