# Supplementary material for: Biofilm Formation Mechanisms of Pseudomonas aeruginosa Predicted via Genome-Scale Kinetic Models of Bacterial Metabolism
Source: PLoS Comput Biol. 2015 Oct 2;11(10):e1004452. doi: 10.1371/journal.pcbi.1004452 (PMC4592021; doi:10.1371/journal.pcbi.1004452)
Supplement: S2 Supporting Information — A compressed archive containing MATLAB files to simulate the metabolism of P. aeruginosa under biofilm conditions. (ZIP) [file pcbi.1004452.s002.zip › S2_Supporting_Information/Readme.rtf]

S2 Supporting Information
Biofilm Formation Mechanisms of Pseudomonas aeruginosa Predicted via Genome-Scale Kinetic Models of Bacterial Metabolism
Francisco G. Vital-Lopez, Jaques Reifman*, and Anders Wallqvist 
Department of Defense Biotechnology High Performance Computing Software Applications Institute, Telemedicine and Advanced Technology Research Center, U.S. Army Medical Research and Materiel Command, Fort Detrick, MD, USA

This S2 Supporting Information contains:
1. simulate_paeruginosa_biofilm.m: Matlab program to simulate the metabolism of P. aeruginosa under biofilm growth using gene expression data. Simulation parameters are defined in this file and it calls “pa_biofilm_simulations.m” to run the simulation.
2. pa_biofilm_simulation.m: Matlab program to set up the simulation runs given the model and the parameters defined in “simulate_paeruginosa_biofilm.m”.
3. p_aeruginosa_kinetic_model.m: The kinetic model in Matlab format, i.e., a Matlab function that computes the right-hand-side of the system of ODEs describing the mass balance of the metabolites. 
4. gem_eval_rules.m: Matlab function to compute the overall gene         expression changes associated with each reaction from the measured         gene expression ratios for each gene.
5. my_events_ss.m: Auxiliary function to stop a simulation when it is not converging to a steady state.
6. p_aeruginosa_model.mat: Matlab data file containing a structure (“model”) defining the model of P. aeruginosa metabolic network and a structure (“data”) defining variables required in the simulations. The lower and upper bounds for FBA are provided in the “model” structure.
7. reference_flux.mat: Matlab data file containing the 100 reference flux distributions used in the manuscript simulations.
8. p_aeruginosa_gene_expression_data.mat: Matlab data file containing the processed gene expression data used in the manuscript simulations.
9. beta_cyclic_pathways.mat: Matlab data file containing the parameter beta for cyclic pathways.

These Matlab files are setup to simulate P. aeruginosa's metabolism under biofilm growth. The simulation is carried out running the program "p_simulate_paeruginosa_biofilm.m". Please locate all the Matlab associated files in the same directory or add the appropriate paths.     

The simulation results are return in a structure containing the following fields:
	C: An m by x matrix of the concentration ratios of the m 		metabolites for each of the x reference flux distributions.
DC: An m by x matrix of the derivative with respect to time of the concentration ratios of the m metabolites for each of the x reference flux distributions.
	R: An r by x matrix of the flux ratios of the r 			metabolic reactions for each of the x reference flux 	distributions.

 
System requirements:
MATLAB, The MathWorks Inc., Natick, MA (http://www.mathworks.com/)
COPASI, (http://www.copasi.org)
Note: the Matlab programs were tested only in MATLAB2014

The supplemented script codes and other related files are provided "as is" and "with all faults." We, the authors of the related manuscript, make no representations or warranties of any kind concerning the safety, suitability, lack of viruses, inaccuracies, typographical errors, or other harmful components of the supplemented files. There are inherent dangers in the use of any software, and you are solely responsible for determining whether the supplemented files are compatible with your equipment and other software installed on your equipment. You are also solely responsible for the protection of your equipment and backup of your data, and we will not be liable for any damages you may suffer in connection with using, modifying, or distributing the supplemented files.
