# Supplementary material for: Biofilm Formation Mechanisms of Pseudomonas aeruginosa Predicted via Genome-Scale Kinetic Models of Bacterial Metabolism
Source: PLoS Comput Biol. 2015 Oct 2;11(10):e1004452. doi: 10.1371/journal.pcbi.1004452 (PMC4592021; doi:10.1371/journal.pcbi.1004452)
Supplement: S2 Supporting Information — A compressed archive containing MATLAB files to simulate the metabolism of P. aeruginosa under biofilm conditions. (ZIP) [file pcbi.1004452.s002.zip › S2_Supporting_Information/~WRL2252.tmp]

These Matlab files are setup to simulate P. aeruginosa's metabolism under biofilm growth. The simulation is carried out running the program "p_simulate_paeruginosa_biofilm.m". Please locate all the Matlab associated files in the same directory or add the appropriate paths.     

The simulation results are return in a structure containing the following fields:
	C: an m by x matrix of the concentration ratios of the m 			metabolites for each of the x reference flux distributions.
	DC: an m by x matrix of the derivative with respect to time of 	concentration ratios of the m metabolites for each of the x 	reference flux distributions.
	R: and r by x matrix of the flux ratios of the r 			metabolic reactions for each of the x reference flux 	distributions.

 
System requirements:
MATLAB, The MathWorks Inc., Natick, MA (http://www.mathworks.com/)
COPASI, (http://www.copasi.org)
Note: the Matlab programs were tested only in MATLAB2011

The supplemented script codes and other related files are provided "as is" and "with all faults." We, the authors of the related manuscript, make no representations or warranties of any kind concerning the safety, suitability, lack of viruses, inaccuracies, typographical errors, or other harmful components of the supplemented files. There are inherent dangers in the use of any software, and you are solely responsible for determining whether the supplemented files are compatible with your equipment and other software installed on your equipment. You are also solely responsible for the protection of your equipment and backup of your data, and we will not be liable for any damages you may suffer in connection with using, modifying, or distributing the supplemented files.
