# Supplementary material for: Biofilm Formation Mechanisms of Pseudomonas aeruginosa Predicted via Genome-Scale Kinetic Models of Bacterial Metabolism
Source: PLoS Comput Biol. 2015 Oct 2;11(10):e1004452. doi: 10.1371/journal.pcbi.1004452 (PMC4592021; doi:10.1371/journal.pcbi.1004452)
Supplement: S1 Text — (DOCX) [file pcbi.1004452.s004.docx]

Supporting Information S1 Text for article:

Biofilm formation mechanisms of *Pseudomonas* *aeruginosa* predicted via genome-scale kinetic models of bacterial metabolism

Francisco G. Vital-Lopez, Jaques Reifman, and Anders Wallqvist

Contents

1. *Method to create an ensemble of random reference flux distributions*
2. *Reproducibility of simulation results*
3. *Validation of the kinetic model by predicting essential reactions for planktonic growth overlooked by FBA*
4. *Biomass growth and synthesis of biofilm components are poor predictors of biofilm formation*
5. *Validation of the scoring function to predict biofilm-reducing reactions*
6. *Definition of parameter* β
7. *Method to create an ensemble of random reference flux distributions*

A reference flux distribution that satisfies the mass balance, thermodynamic, and simulation condition constraints can be obtained with the following four-step procedure:

*Step 1*. We computed the lower and upper bounds of every reaction in the network. The lower bound was computed by solving the following optimization problem:

|  | $\begin{matrix} \min v_{i} \\ \text{s.t.} \\ (\text{S1A}) \mathbf{S}\cdot\mathbf{v}=\mathbf{0} \\ (\text{S1B}) v_{i}\geq0 \forall i\in IR \\ (\text{S1C}) v_{oxy}\leq w_{oxy}\cdot v_{oxy}^{opt} \\ (\text{S1D}) x_{j}v_{j}\leq w_{CS}\sum_{i\in CS} x_{i}v_{i} \forall j\in CS \\ (\text{S1E}) \sum_{i\in CS} x_{i}v_{i}\leq2Y^{opt} \\ (\text{S1F}) v_{biomass}=1 \end{matrix}$ | (S1) |
| --- | --- | --- |

where *v_i_* is the flux of reaction *i*, **S** is the stoichiometric matrix, **v** is a vector of elements *v_i_*, and *IR* is the set of irreversible reactions. The variable *v_oxy_* is the oxygen uptake rate, $v_{oxy}^{opt}$is the optimal oxygen uptake rate (i.e., to maximize the biomass yield), and *w_oxy_* is a constant with values of 1.00 and 0.20 for exponential and stationary planktonic culture conditions, respectively. The coefficient *x_i_* is the carbon content in metabolite *i*, *CS* is the set of metabolites that can be used as a carbon source, and the constant $w_{CS}$has the values of 1.00 and 0.25 for exponential and stationary planktonic culture conditions, respectively. The coefficient$Y^{opt}$ is the total carbon uptake at optimal biomass yields and $v_{biomass}$ is the biomass growth rate. This problem determines the lower bound of reaction *i*. S1A and S1B are the mass balance and thermodynamic constraints. S1C constrains the oxygen uptake rate, and S1D constrains the fraction of carbon from a single source depending on the simulation condition. S1E constrains the total carbon uptake to be within a factor of 2 of the carbon uptake at the optimal biomass yield, and S1F sets the biomass growth rate to 1. To compute the upper bound, the objective function is maximized instead.

Some of the reactions may have an unbounded lower bound, unbounded upper bound, or both. This generally occurs when reaction cycles are present in the metabolic network. To compute meaningful lower and upper bounds for an unbounded reaction, we constrained the flux of one of the reactions in a cycle to zero and computed the lower and upper limits for the rest of the reactions in the cycle. We took the lowest and largest values of each reaction from these calculations as the lower and upper bounds for the unbounded reactions, respectively.

*Step 2*. We generated 100 random flux vectors by sampling the space constrained by the lower and upper bounds of each reaction computed in *step 1*. We sampled this space using a Latin hypercube design.

*Step 3*. The random flux vectors generated in *step 2* do not satisfy the constraints of problem S1. Therefore, for each random flux vector, we determined a flux vector that was as close as possible to it while satisfying the constraints in problem S1:

|  | $\begin{matrix} \min\sum_{i} \left\vert v_{i}-v_{i}^{rand}\right\vert\\ \text{s.t.} \\ \mathbf{S}\cdot\mathbf{v}=\mathbf{0} \\ v_{i}\geq0 \forall i\in IR \\ v_{oxy}\leq w_{oxy}\cdot v_{oxy}^{opt} \\ x_{j}v_{j}\leq w_{CS}\sum_{i\in CS} x_{i}v_{i} \forall j\in CS \\ \sum_{i\in CS} x_{i}v_{i}\leq2Y^{opt} \\ v_{biomass}=1 \end{matrix}$ | (S2) |
| --- | --- | --- |

where$v_{i}^{rand}$is the flux of reaction *i* in the random flux vector.

The solution of problem S2 may include of the reactions with zero flux, even if their lower or upper bounds are non-zero. However, we assumed that if the enzyme(s) and substrate(s) of a given reaction are present, then the reaction must have a non-zero flux. To compute a non-zero flux for such reactions, we solved the following problem:

|  | $\begin{matrix} \min\sum_{i\in Z} \left\vert v_{i}-v_{i}^{rand}\right\vert\\ \text{s.t.} \\ \mathbf{S}\cdot\mathbf{v}=\mathbf{0} \\ v_{i}\geq0 \forall i\in IR \\ v_{oxy}\leq w_{oxy}\cdot v_{oxy}^{opt} \\ x_{j}v_{j}\leq w_{CS}\sum_{i\in CS} x_{i}v_{i} \forall j\in CS \\ \sum_{i\in CS} x_{i}v_{i}\leq2Y^{opt} \\ v_{biomass}=1 \\ \sum_{i} \left\vert v_{i}-(v_{i}^{l}+v_{i}^{u})/2\right\vert\leq z_{0}+L \end{matrix}$ | (S3) |
| --- | --- | --- |

where *Z* is the set of reactions with zero flux,$z_{0}$is the objective function value for the solution of problem S2, and *L* is a slack parameter. Problem S3 was solved iteratively until all possible reactions had a non-zero flux. The reference flux distribution was then computed from all solutions as follows:

|  | $\mathbf{v}^{ref}=\frac{1}{m}\sum_{j} \mathbf{v}_{j}$ | (S4) |
| --- | --- | --- |

where *m* is the number of solutions **v***_j_*.

1. *Reproducibility of simulation results*

We carried out all analysis based on the distributions of metabolite concentration and flux changes obtained from simulations using 100 random reference flux distributions to account for the uncertainty of these parameters. We checked if the sample size we used was sufficient to obtain a reproducible estimate of the distributions of the metabolite concentration and flux changes with two tests. In both tests, we used the predicted metabolic changes between biofilms and stationary planktonic cultures. First, we randomly divided the 100 simulations into 2 sets of 50. We then compared the distributions of the metabolite concentration and flux changes derived from each set. Significant differences between the distributions from the two sets are an indication of an insufficient number of simulations. We estimated the significance of the difference between distributions using the Kolmogorov-Smirnov test. This nonparametric hypothesis test compares the cumulative distribution functions of the two samples to determine if the samples were drawn from the same distribution (null hypothesis) or from different distributions (alternative hypothesis). After repeating the test 100 times, we found that only for ~5% of the metabolites and 4% of the reactions the null hypothesis was rejected at a 5% significance level (which is around the expected percentage of null hypothesis rejections at this significance level when the samples are drawn from the same distribution).

We then checked how much the estimated mean and standard deviation of the distribution of the metabolite concentration and flux changes varied with the number of simulations. For this, we computed the means and standard deviations of the concentration and flux changes by randomly selecting 50 simulations and compared them with the means and standard deviations estimated with the 100 simulations. We found that the means of the concentration and flux changes estimated with 50 simulations were within 5% from the means estimated with the 100 simulations for 98% of the metabolites and 96% of the reactions, respectively. As expected, the standard deviations had more variation than the means. The standard deviations of the concentration and flux changes estimated with 50 simulations were within 20% of the standard deviations estimated with the 100 simulations for 94% of both the metabolites and reactions. The results of these two tests suggest that reproducible results can be obtained even with 50 simulations of these conditions.

1. *Validation of the kinetic model by predicting essential reactions for planktonic growth overlooked by FBA*

As a validation of our approach, we tested whether the kinetic model could predict additional important reactions for biomass growth that would be overlooked using a conventional FBA approach. Note that in this section, we focused on the exponential growth phase of planktonic cultures (exponential cultures) because the essential genes were identified under such conditions [[1](#_ENREF_1), [2](#_ENREF_2)]. First, we identified essential reactions for growth in Luria-Bertani medium using the FBA approach. We predicted 185 of 659 reactions as essential for biomass growth in Luria-Bertani medium (26 reactions representing drain fluxes of biomass precursors and the biomass growth reaction were not considered). The list of predicted essential reactions is provided in S1 Table. Eighty-seven of the predicted essential reactions were associated with genes from the set of experimentally determined essential genes for biomass growth (referred to as “essential genes” hereafter) compiled by Sigurdsson et al. [[3](#_ENREF_3)]. Of the predicted 474 non-essential reactions, 416 reactions were associated only with non-essential genes. In total, the model had 145 reactions associated with essential genes. Thus, the FBA-based approach yielded a sensitivity of 0.60 (87/145) and an accuracy of 0.76 [(87 + 416)/659], which were in line with previous FBA results of the *P. aeruginosa* metabolic network based on the inhibition of single genes instead of reactions [[3](#_ENREF_3), [4](#_ENREF_4)]. This set of predicted reactions was essential for both exponential and biofilm cultures, in agreement with previous FBA work that reported no biofilm-specific essential reactions [[3](#_ENREF_3)].

Next, we predicted that 6 of the top 20 reactions with a higher effect on the biomass growth rate were associated with an essential gene (see S2 Table). We computed the probability of over-representation of correctly predicted reactions among the selected reactions using a hypergeometric distribution. The hypergeometric distribution describes the probability of getting *k* objects with a given attribute in a random sample of *n* objects drawn from a population of *N* objects containing *K* objects with a specific attribute. The population of reactions that had gene associations and were predicted non-essential by FBA had 433 reactions, including 58 reactions associated with an essential gene. Thus, the probability (*p*) of getting 6 or more reactions associated with an essential gene in a random sample of 20 reactions drawn from this population is given by the complement of the following hypergeometric cumulative distribution function:

|  | $p=1-\sum_{i=0}^{6-1} \frac{\left(\begin{matrix} 58 \\ i \end{matrix}\right)\left(\begin{matrix} 433-58 \\ 20-i \end{matrix}\right)}{\left(\begin{matrix} 433 \\ 20 \end{matrix}\right)}=0.04$ | (S5) |
| --- | --- | --- |

1. *Biomass growth and synthesis of biofilm components are poor predictors of biofilm formation*

Similar to the exponential cultures, we used the kinetic model to simulate the effect of blocking the FBA-predicted non-essential reactions under biofilm conditions and ranked the reactions according to the effect of their inhibition on the biomass growth rate. None of the top 20 reactions was associated with an essential gene (see S2 Table). This result was not surprising, because the essential genes were identified under planktonic conditions [[1](#_ENREF_1), [2](#_ENREF_2)]. However, none of the predicted top 20 reactions was associated with a gene determined to be important for biofilm formation in a genome-wide screen using a library of transposon mutants of the *P. aeruginosa* strain PA14 [[5](#_ENREF_5)]. Actually, two of the reactions (glyceraldehyde-3-phosphate dehydrogenase and pyruvate dehydrogenase) were associated with genes (*gapA* and *aceE*, respectively) whose mutations induced higher biofilm formation than the wild-type strain. This result suggests that biomass growth may be a poor predictor of biofilm formation. In fact, Musken et al. [[5](#_ENREF_5)] found slow-growing mutants in both low and high biofilm-producing strains.

We also tested if inhibition of the synthesis of the major biofilm components (i.e., the exopolysaccharides Psl and Pel, DNA, and proteins) would predict mutants with reduced biofilm formation. We predicted 36 reactions whose inhibition would reduce the synthesis rate of at least one of the major biofilm components by a factor of 2, but only 3 reactions of them were in the set of 78 reactions associated with experimentally determined biofilm-reducing mutants. This result shows that a scoring function based on the synthesis rate of the major biofilm components is also a poor predictor of biofilm formation.

1. *Validation of the scoring function to predict biofilm-reducing reactions*

To validate the proposed scoring function, we examined the literature for additional biofilm-defective mutants that were not included in the group of mutants identified by Musken et al. [22]. We found 15 additional reactions that were associated with low biofilm-producing mutants (S4 Table), of which 8 reactions were in the set of reactions whose inhibitions we predicted to reduce biofilm formation. As in the case of the predictions of essential reactions, we used the hypergeometric distribution to estimate the probability of over-representation of correctly predicted biofilm-reducing reactions. In this case, we had a population of 547 reactions, including 15 reactions associated with known low biofilm-producing mutants. We picked 8 of these 15 reactions in the set of the 36 predicted biofilm-reducing reactions. Thus, the probability of getting eight or more biofilm-reducing reactions in this scenario is given by the complement of the following hypergeometric cumulative distribution function:

|  | $p=1-\sum_{i=0}^{8-1} \frac{\left(\begin{matrix} 15 \\ i \end{matrix}\right)\left(\begin{matrix} 547-15 \\ 36-i \end{matrix}\right)}{\left(\begin{matrix} 547 \\ 36 \end{matrix}\right)}=0.02$ | (S6) |
| --- | --- | --- |

1. *Definition of parameter* β

Parameter β depends on the equilibrium constant and the concentration of substrates and products at the reference condition. The following problem describes a reversible reaction:

|  | $\sum_{ i} a_{i}A_{i}\leftrightarrow\sum_{j} b_{j}B_{j}.$ | (S7) |
| --- | --- | --- |

The relation between *β* and the equilibrium constant *K_eq_* is given by the following:

|  | $\beta=\frac{1}{1-Q/K_{eq}},\text{ if }v^{ref}>0,$ | (S8) |
| --- | --- | --- |
|  | $\beta=\frac{1}{1-K_{eq}/Q},\text{ if }v^{ref}<0,$ | (S9) |
|  | $K_{eq}=\prod_{j} [B_{j}^{eq}]/\prod_{ i} [A_{i}^{eq}],$ | (S10) |
|  | $Q=\prod_{j} [B_{j}^{ref}]/\prod_{ i} [A_{i}^{ref}],$ | (S11) |

where *A_i_*, *B_j_*, *a_i_*, and *b_j_* are substrate *i*, product *j*, and their corresponding stoichiometric coefficients, respectively. The square brackets indicate concentrations, and the superscripts *eq* and *ref* indicate equilibrium and reference conditions, respectively.

From eqs. S8 and S9, we note that *β* increases as the reaction approaches equilibrium (i.e., *K_eq_*/*Q* → 1.0). Thus, a large value of *β* corresponds to a near-equilibrium reaction and a small value of *β* corresponds to a far from equilibrium reaction.

**References:**

1. Jacobs MA, Alwood A, Thaipisuttikul I, Spencer D, Haugen E, Ernst S, et al. Comprehensive transposon mutant library of *Pseudomonas aeruginosa*. Proc Natl Acad Sci U S A. 2003 Nov 25;100: 14339-14344.

2. Lewenza S, Falsafi RK, Winsor G, Gooderham WJ, McPhee JB, Brinkman FS, et al. Construction of a mini-Tn5-luxCDABE mutant library in *Pseudomonas aeruginosa* PAO1: a tool for identifying differentially regulated genes. Genome Res. 2005 Apr;15: 583-589.

3. Sigurdsson G, Fleming RM, Heinken A, Thiele I. A systems biology approach to drug targets in *Pseudomonas aeruginosa* biofilm. PLOS ONE. 2012;7: e34337.

4. Oberhardt MA, Puchalka J, Fryer KE, Martins dos Santos VA, Papin JA. Genome-scale metabolic network analysis of the opportunistic pathogen *Pseudomonas aeruginosa* PAO1. J Bacteriol. 2008 Apr;190: 2790-2803.

5. Musken M, Di Fiore S, Dotsch A, Fischer R, Haussler S. Genetic determinants of *Pseudomonas aeruginosa* biofilm establishment. Microbiology. 2010 Feb;156: 431-441.
