# Supplementary figures and images for: Biofilm Formation Mechanisms of Pseudomonas aeruginosa Predicted via Genome-Scale Kinetic Models of Bacterial Metabolism
Source: PLoS Comput Biol. 2015 Oct 2;11(10):e1004452. doi: 10.1371/journal.pcbi.1004452 (PMC4592021; doi:10.1371/journal.pcbi.1004452)

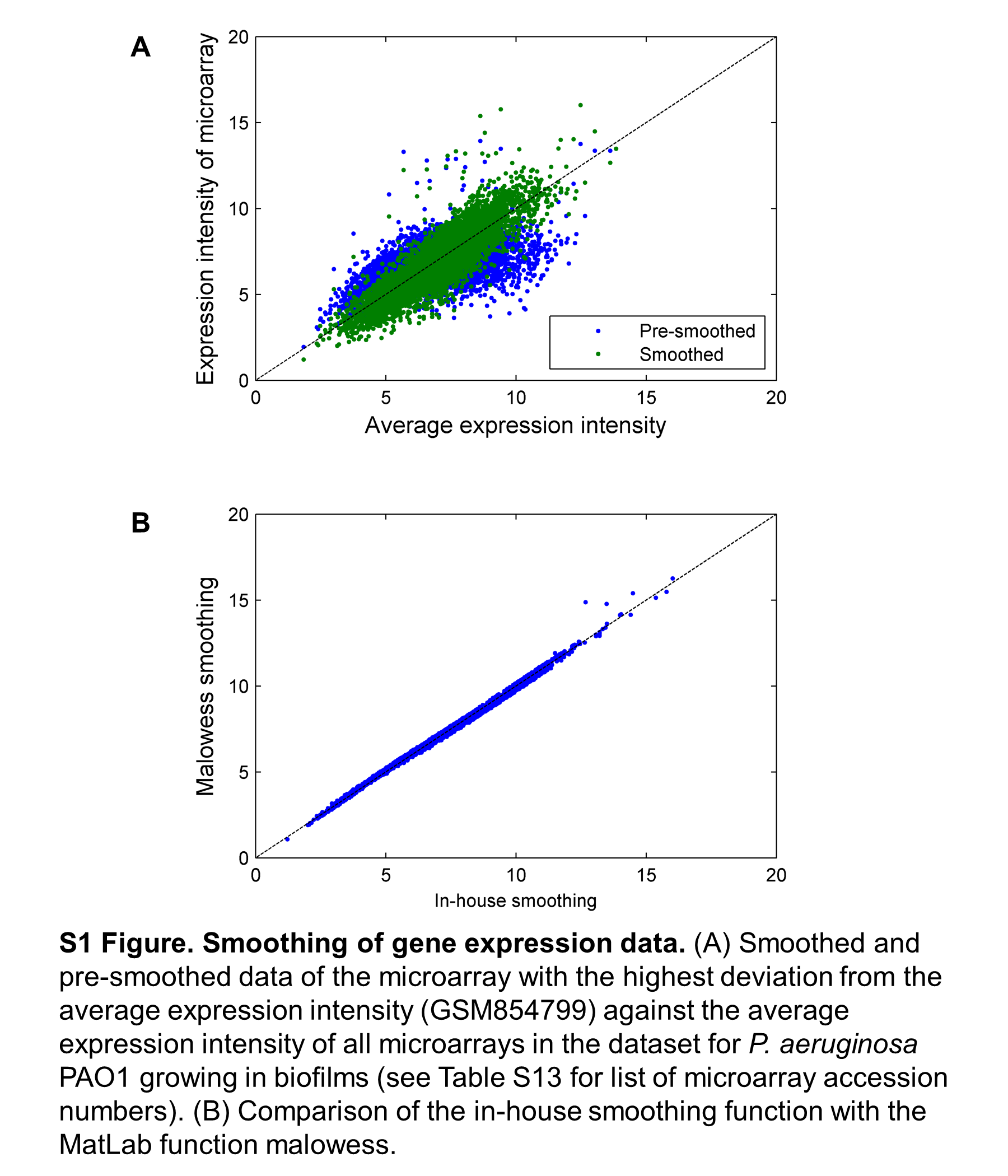

Supplement: S1 Fig — (A) Smoothed and pre-smoothed data for the microarray with the highest deviation from the average expression intensity (GSM854799), compared to the average expression intensity of all microarrays in the dataset for P. aeruginosa PAO1 growing in biofilms (see S13 Table for microarray accession numbers). (B) Comparison of the microarray data smoothed using the in-house-developed smoothing function and the MATLAB function malowess. (TIF) [file pcbi.1004452.s018.tif]
